# Reviews of Books

**Published:** 1924-05

**Authors:** 


### REVIEWS OF BOOKS.

### A PIONEER MEDICAL WOMAN.

**Reminiscences.** By Mary Scharlieb, M.D., M.S. (Williams and Norgate. 12s. 6d. net.)

As a pioneer medical woman, Mrs. Scharlieb has a stirring story to tell. She has told it, she modestly says, " not for my own sake, not even for the joy that it has brought me. I hope that my sense of my own insignificance would have made me feel that it was foolish to add yet one more to the many Reminiscences recently published, most of which were framed in a more interesting manner and had better claims on the attention of the public. But my object is to convince medical women students and junior practitioners that a successful, happy, and useful career can be, and ought to be, the guerdon of their toil, though, inasmuch as we can never get more out of any enterprise than we put into it, they are likely to find that success and opportunities of usefulness will vary directly with the vigour that they put into their studies and the love that they bring into professional practice." Mrs. Scharlieb has amply fulfilled her aim, and she has in addition written a book of peculiar interest which will be widely read.

It was during her early married life in India that she heard of the suffering undergone by native women in time of sickness and child birth, and it was the desire to help these unfortunate creatures that led her finally into the paths of medicine :—

A very young Mahommedan lady, a child herself, had a difficult and extremely painful labour. Her husband, although a strict Mahommedan and jealously observant of *purdah* rules, was finally so affected that he sent to a European surgeon to come to try to save her life. The poor girl dragged herself to the door of her room and lay down against it so that it could not be opened from without except by brutally crushing her body. Her women approved of her action and certainly did not dare to touch her, and there she lay until death ended her sufferings.

Qualifying at Madras Medical College, she worked for some time in India, but her health and her children's schooling necessitated a return home, and she studied for her M.B. at the newly founded School of Medicine for Women :—

I was so fortunate as to get honours all round with the Gold Medal and Scholarship in Obstetric Medicine. The examiners in this subject were the late Matthews Duncan and the late Dr. Gervis. Matthews Duncan had the character of being a real old bear, but he was extremely kind, although somewhat gruff in manner, and subsequently he told a friend that he " liked a candidate who made no effort to conceal her ignorance." From the son of an old friend, who was one of Dr. Gervis's students at St. Thomas's Hospital, I heard that when he learnt that No. 104 was a woman, Dr. Gervis exclaimed : " Oh ! to think that I have lived to give the scholarship in my own subject to a woman ! But there, it was an excellent paper and it can't be helped ! "

More work at Madras followed, but in 1887 Mrs. Scharlieb's bad health compelled her to return finally to England, and she had then before her the hard task of building up a practice in London. Her children still needed care and she possessed no capital, yet a comparatively short time saw her well established, and for over thirty years she has been perhaps the best known woman doctor in this country. " The professional prospects of women are," she thinks, " decidedly good " :—

The powers that be have gradually learned that women doctors can be relied on to do certain kinds of work and to them well. The record of the women's medical work during the war affords admirable proof of their professional and administrative ability; it also shows that they are good colleagues and cheery comrades. The story of their doings is not only interesting, but it goes far to prove their value and to justify their existence. Medical women are being more and more utilised in the public services.

But Mrs. Scharlieb is not ashamed of being thought old-fashioned, and her conviction is that " women doctors will succeed or fail according to their merits, and that those merits must be sought not only in the intellectual and professional, but still more in the moral and spiritual status they may gain."

### VITAMINS REVIEWED.

**Medical Research Council, Special Report Series,** No. 38, Revised. (H.M. Statonery Office. 4s. 6d. net.)

No one doubts the immense importance of the discovery of vitamins, but, as so often happens with recent advances in medical science, it is possible that some of the earlier claims went a little too far, and that—particularly in commercial exploitation of the vitamins theory—unduly optimistic statements were made. Therefore, all will welcome the appearance of a revised edition of the Medical Research Council's Report on the present state of our knowledge of these necessary food factors.

Since the original report was published considerable progress has been made in determining the exact origin and distribution of vitamins, and, what is even more important, the conditions which govern their stability. Also, many directly medical observations have tested, at the bedside or in the clinic, the earlier vitamin theories. In spite of this two striking facts remain. As yet vitamins have not been isolated in the pure state, and we still, in spite of much effort, remain almost completely ignorant of how these substances exert their influence on the body. In such circumstances we must conclude that the present Report will require before long further revision, and possibly that some of these latest deductions should be accepted with a certain reserve.

Nevertheless, there are several phases of the newer work which have so direct a bearing upon everyday life and are, or appear to be, now so well established that there is every justification for doubly impressing them upon the public mind. Not the least of these is the latest research conclusions upon rickets. Two outstanding factors are concerned in protection from this disease, an anti-rachitic vitamin and sunlight. Both these are probably concerned in the proper deposition of calcium and phosphorus during the growth of cartilage and bone. Cod liver oil is the richest known source of anti-rachitic vitamin ; milk, eggs and butter rank next among the commonly available substances. Sunlight and other forms of light radiation have been shown to possess preventive and curative effects in rickets comparable in many respects with those of cod liver oil. Sunlight will even make safe some diets which are relatively deficient in the vitamin.

Therefore it is, as the Report explains, now intelligible why rickets is a comparatively rare disease both in natives living under tropical conditions and in the inhabitants of the Arctic Circle. The extra sunshine enjoyed by the one set of people makes the smaller amount of calcifying vitamin in their diet do the work carried out by the larger amount received by the Esquimo. Now there is little doubt that town dwellers of the temperate zones who live under modern industrial conditions are very liable to receive a poor supply of both light and vitamin, and attention to this point must be the key to rickets prevention. With regard to retaining the vitamins naturally formed in our food, slow cooking or steaming is, generally speaking, to be condemned. It certainly is as far as vegetables are concerned, and various outbreaks of "deficiency disease" have been clearly traced to forgetfulness of this fact.

Another point to be clearly emphasised is the importance of breast-feeding. It is probably impossible to elaborate a perfect substitute for mother's milk, the nutritional advantage of which must, apart from simpler chemical constitution, depend upon an appropriate mixture of vitamins unweakened by heat, oxidation or dilution. It is, too, vitally important to remember that the mother is entirely dependent upon her own food supply and sunlight to provide her children with the necessary vitamins. In this country there is little chance of mother's milk being, from this cause, totally deficient in any one of the accessory food factors, but the risk is certainly present in, for example, such a tendency as the increasing substitution of cheap margarine for butter. It is, in fact, the appearance upon the market of a great variety of prepared milks, preserved foods and other substitutes which makes the facts reviewed in this Report invaluable to the hygienist. Let us note the findings as concerns the first. Dried milks prepared by the "drum" process, in which the exposure to heat is very short, lose, probably, very little active vitamin. Somewhat more is destroyed in those produced by the "spraying" process. But in either case recent experiments suggest that all dried milks tend to lose vitamins by slow oxidation if they are kept for long periods before being used. Therefore, although dried milks possess many advantages for use in densely populated areas and provide a most satisfactory method of keeping good clean milk available for everyone, this opportunity for storage and slower transport must not be abused, or all the advantages may be lost by the time the milk is consumed.

Concerning infants' foods it is to be noted that, apart from those made directly from cow's milk, the majority consist largely of cereals, frequently malted. Most of these lack fat, and consequently the fat-soluble vitamins also, when given alone. Apparently some of these are also inadequate as regards the other vitamins, and it is evident that even the addition of fresh milk to such foods will inevitably produce a mixture which is inferior, as far as vitamins are concerned, bulk for bulk, to fresh mother's milk. This fact constitutes the most powerful argument against extensive use of these foods in infant feeding. Similarly, reading through this Report, we might collect whole series of simple but all-important points to which popular attention ought most certainly to be drawn, and we hope that those to whose lot it falls to convey to the public the true import of these scientific advances will study in detail its interesting and carefully compiled chapters. Their labour will be not in vain.

### ARMS AND THE WOMAN.

**The Bearing of Coat-Armour by Ladies.** By Charles A. H. Franklin, F.S.A., M.R.C.S., L.R.C.P. (Murray. 12s. net.)

Mr. Franklin is that unusual combination, a doctor and an accomplished herald. He has written an admirable book upon a department of heraldry which is, perhaps, even less understood than other departments of a science upon which a hasty and indifferent world is content to remain lamentably ignorant, and if we are not always in agreement with him that is because he claims too much for official armory, and is, apparently, content that it should continue unreformed and unprogressive. We are glad to see that, at the outset, he lays stress upon the fundamental fact that the only nobility is that of coat-armour—the farm labourer who is entitled to a coat of arms if only he knew it is as much a noble as the Duke of Norfolk. This is well understood on the Continent, but has been lost sight of in England.

Starting from this bedrock of fact it is obvious that the usurper or inventor of heraldic emblems is in the same position as a man who should pretend to be a peer, or a parson, or a medical man who cannot produce his *parchemins*. But Mr. Franklin, like many of the school of thick and thin advocates of the College of Arms, uses very hard language about the impostors, many of whom are perfectly innocent of evil intent and are merely doing as their fathers and grandfathers did. The contention of this school is that arms not registered at Heralds' College are necessarily "bogus." That is a claim which cannot be admitted, as Dr. J. H. Round, one of our most distinguished authorities, has shown. It is inevitable that through centuries of history and civil disturbance there should have been lapses, and one of the heraldic reforms badly needed is the introduction in England of a recognised and cheap system of confirmation of unregistered arms such as exists in Ireland.

Upon the main theme of his book, the inheritance, use, and transmission of arms by women, Mr. Franklin has much that is interesting to say. Women bear their family heraldry, not upon a shield, but a lozenge, and they cannot use a crest for the sufficient reason that they have no helmet upon which to wear it. These simple rules are often ignored, with the result that the poor ignorant ladies are sometimes made to look ridiculous. Mr. Franklin emphasises the strict letter of heraldic law, that a woman cannot transmit her personal arms to her descendants unless she is an heiress or a co-heiress. This is the merest anise and cummin, and we should like to see the rise of a bold and reforming school of heralds who would teach that the blood carries the arms, heiress or no heiress. We are well aware that this is rank blasphemy, and that every Herald and Pursuivant in Queen Victoria Street will be speechless with horror at this rreverent laying of hands upon the ark of the covenant. But if heraldry is to be a living science it must be modernised. Nor do we see any valid reason why a woman, although she cannot wear a crest, should not transmit it, on the ground that it is merely in abeyance during her lifetime.

That heraldic usage is capable of modernisation and has, in fact, been modernised, one instance will suffice to show. We poke fun at the Chinese for ennobling their ancestors, but we do the same thing ourselves. The convenient and entirely laudable practice has grown up of making grants of arms to include not only the grantee, but the other descendents of his father, and even his grandfather. Moreover, we now confer nobility upon American citizens by granting them "Honorary armorial bearings." Here are two innovations. Why, then, not those also that we have suggested, and perhaps others ? A grant for £25 (instead of nearly £80) and a confirmation for £10 would enable many "bogus" users of arms to regularise their position. Mr. Franklin appeals to women to help to keep heraldry pure; we would appeal to them to help to bring the gentle science of blazon more closely into accord with modern ideas. For the matter of that, it is more than a science. It is a highly decorative art which, in its love of colour, should touch a responsive chord in the feminine bosom. Meanwhile, Mr. Franklin has done the sex a service by instructing it upon the proper use of its armorial insignia. Also he has provided a highly stimulating book, full of the pure milk of the heraldic word, a book which is lavishly and beautifully embellished in black and white and in colour.

The one thing that astonishes us is that a purist like Mr. Franklin should speak of something as "rather unique." Were we to describe a gentleman of coat-armour as "rather noble" we should expect to be cited before the Earl Marshal's Court, with Mr. Franklin in attendance as Lord High Torturer. Yet he has done a real service to a noble and historic subject, and his book will assuredly take a distinguished place in modern heraldic literature.

---

### MISCELLANEOUS NOTICES.

### Diabetes and Insulin.

Modern Methods in the Diagnosis and Treatment of Glycosuria and Diabetes. By Hugh MacLean, M.D., D.Sc. Second Edition, revised and enlarged. (London: Constable. 12s. net.)—The early appearance of the second edition of this excellent monograph is sufficient indication of the great service it has been to the practising medical profession. In so short a time relatively little change has occurred in the general situation concerning insulin, but the author takes the opportunity of once more impressing upon medical men the urgent necessity of making themselves familiar with the general principles involved in its use. The fact remains that patients are still dying from diabetic coma, without any attempt being made to save them by the use of insulin—a condition of affairs for which, as the author says, there is now really no excuse.

### A Text-Book on Bacteriology.

Elementary Bacteriology for Nurses. By G. Norman Meachen, M.D., B.S.(Lond.), etc. Second Edition. (The Scientific Press. 3s. net.)—Sister-tutors and others charged with the responsibility of preparing student-nurses for the State Examination will be glad to see a second edition of Dr. Meachen's very lucidly-written little book, which makes interesting what is looked upon as rather a "dry" study, and, moreover, embraces all the knowledge required by the State Syllabus from nurses on the subject of bacteriology. To this edition, which has been brought quite up to date, and has in many parts been wholly rewritten, are added many interesting new paragraphs on spirochætal injections, encephalitis lethargica and other subjects which have lately sprung into greater prominence owing to the researches and advances of science since the book first appeared in 1914. Its usefulness has therefore increased, and it should continue to be a valuable text-book on bacteriology for nurses for some years to come.

### Medicine and the Mind.

Medicine and the Mind. By Giles F. Goldsbrough, M.D. (John Bale, Sons & Danielsson.)—This pamphlet is an expansion of a post-graduate lecture. The title might indicate a treatise on psycho-analysis, suggestion, or any similar topic relating to mental therapeutics. But this is not the object of the pamphlet. Dr. Goldsbrough's desire has been to give a philosophical introduction to the subject of medicine in relation to the mind. It is, no doubt, much to be wished that medical practitioners should have some acquaintance with philosophy. Such acquaintance might tend to make them realise the inevitable limitations of our knowledge, and to make them pause before confidently assigning "causes" for diseases and other phenomena. No adequate introduction can possibly be given to this vast subject within the limits of a small pamphlet, a fact which the author would be the first to admit. But it is quite possible that some readers may be stimulated to make further inquiry. In addition to philosophy, Dr. Goldsbrough gives us some excellent reflections upon the high ideals which are necessary for a hospital physician, and the absolutely disinterested devotion to treatment and research which should be possessed by him.

### A Synopsis of Obstetrics.

Synopsis of Midwifery. By A. C. Magian, M.D.. Gynæcologist to the Manchester French Hospital, etc. (William Heinemann: Medical Books, Ltd. Price 8s. 6d, net.)—This "Synopsis" is what it professes to be—"a general view of the subject of midwifery with its parts so arranged as to display the whole of the principal points." In it all the leading facts and principles of treatment are clearly and concisely stated, and the matter is enriched by reference to the opinions of many well-known writers on the subject. The last five chapters are concerned with obstetrical operations and are very definite and helpful in their instructions, the indications for each being carefully tabulated for ready reference in an emergency and the methods briefly described. A useful list is also given of the necessary instruments required in each operation. Much information has been compressed into the comparatively small space of 245 clearly-printed pages, including a good index, and the necessarily sparing use of words makes for clarity of thought and rapid mental assimilation by the student. As a "refresher" in memorising leading principles in obstetrics the book should be of value, not only for examination purposes, but also to the general practitioner in his daily rounds. One other point to note is that its moderate price places it within the reach of all.

### A Novel of Character.

The Second Wife. By Lilian Arnold. (Thorntou Butterworth. 7s.)—Mrs. Arnold is making rapid strides as a novelist of character, and "The Second Wife" is a long advance upon her previous work. There is distinct originality in the plot, which, simple as it is, is worked out with a naturalness that is easy and inevitable. The two men of the book are both doctors, but while the one is a Harley Street specialist, his brother is an unhappy man who had been involved in a terrible tragedy which had ruined his life. To them enter two women as strongly contrasted as the two brothers, and they it is who make the strength of the book. The girl of the simple, elemental type sets out to be the saviour of a wrecked life; the other, more detached, glorying in her independence, full of vigour of character, language and action, is the stronger type, and is drawn with a surety that makes her live. It is, in short, an extremely human story, in which the characterisation is powerfully helped by the admirable dialogue, which flows with delightful ease. It is true that the heroine almost makes another tragedy in a tragic life, but that is logically in accord with her character, and there is nothing conventional in the happy ending of a story that is well conceived and told with the artlessness of true art.

